# Expression of CYP24A1 and other multiple sclerosis risk genes in peripheral blood indicates response to vitamin D in homeostatic and inflammatory conditions

**DOI:** 10.1038/s41435-021-00144-6

**Published:** 2021-06-23

**Authors:** Samantha P. L. Law, Prudence N. Gatt, Stephen D. Schibeci, Fiona C. McKay, Steve Vucic, Prue Hart, Scott N. Byrne, David Brown, Graeme J. Stewart, Christopher Liddle, Grant P. Parnell, David R. Booth

**Affiliations:** 1 Centre for Immunology and Allergy Research, Westmead Institute for Medical Research, University of Sydney, Sydney, NSW Australia; 2 Telethon Kids Institute, Perth Children’s Hospital, Perth, WA Australia

**Keywords:** Autoimmunity, Gene expression, Inflammation, Gene regulation in immune cells

## Abstract

Although genetic and epidemiological evidence indicates vitamin D insufficiency contributes to multiple sclerosis (MS), and serum levels of vitamin D increase on treatment with cholecalciferol, recent metanalyses indicate that this vitamin D form does not ameliorate disease. Genetic variation in genes regulating vitamin D, and regulated by vitamin D, affect MS risk. We evaluated if the expression of vitamin D responsive MS risk genes could be used to assess vitamin D response in immune cells. Peripheral blood mononuclear cells (PBMCs) were isolated from healthy controls and people with MS treated with dimethyl fumarate. We assayed changes in expression of vitamin D responsive MS risk (VDRMS) genes in response to treatment with 25 hydroxy vitamin D in the presence or absence of inflammatory stimuli. Expression of CYP24A1 and other VDRMS genes was significantly altered in PBMCs treated with vitamin D in the homeostatic and inflammatory models. Gene expression in MS samples had similar responses to controls, but lower initial expression of the risk genes. Vitamin D treatment abrogated these differences. Expression of CYP24A1 and other MS risk genes in blood immune cells indicate vitamin D response and could enable assessment of immunological response to vitamin D in clinical trials and on therapy.

## Introduction

Many of the most common autoimmune diseases show a latitude dependence: prevalence increases with distance from the equator in genetically similar populations in many countries [1–4]. These latitude-dependent autoimmune diseases (LDADs) include multiple sclerosis (MS), type 1 diabetes, rheumatoid arthritis, Crohn’s disease, systemic lupus erythematosus, and psoriasis [5]. The effect can be very strong, e.g., MS is seven times more common in Tasmania than Northern Queensland in Australia [6]. Since the main source of vitamin D is the conversion of 7 dehydrocholesterol to pre-vitamin D in the skin by the action of UVB from sunlight, and deficiency of vitamin D is also associated with increased MS risk independently of sun exposure [7], vitamin D has been suggested as mediating the latitude effect.

LDADs result from the interaction of environmental factors with a complex genetic predisposition. Much of the heritability has now been explained by genome-wide association studies (GWAS) [8–10]. Identified genes affecting pathogenesis in MS include the two genes controlling vitamin D activation and inactivation: CYP27B1 and CYP24A1 [11]. In addition, many of the other MS and other LDAD risk genes are regulated by the vitamin D receptor (VDR) [12]. The recent and expanding understanding of the importance of vitamin D in immune tolerance [13, 14], the role of vitamin D deficiency in MS risk, and the over-representation of genes that control vitamin D metabolism and effect as part of the genetic architecture of LDADs, establish vitamin D-influenced immune pathways as important targets for translational research.

However, despite this evidence, vitamin D supplementation has been of equivocal benefit in clinical trials for LDADs [15, 16], even with supplementation clearly increasing serum 25(OH)D3 levels. This failure of clinical response to vitamin D suggests immunological changes are not ameliorated by increasing serum 25(OH)D3. Vitamin D preparations most used in clinical trials, and widely to treat MS, are two inactive forms, cholecalciferol and ergocalciferol [17]. Some 3000 other pharmacological forms of vitamin D are available, and the tolerogenic effects of the vitamin D response pathway may be more tractable through these or other approaches. Unfortunately, no clinical measure of immunological changes in response to vitamin D supplementation has been established, which limits investigations into the utility of other approaches to manipulating the vitamin D response pathway.

The classical role of vitamin D is to activate the VDR, a transcription factor, indicating vitamin D confers protection from autoimmune diseases through the genome-wide binding sites of the VDR in the many cell types in which it functions, controlling the interaction between and amongst these cell types. For activation, pre-vitamin D from the skin needs to be hydroxylated to 25(OH)D3 in the liver (by MS risk gene CYP2R1 product) and in the skin, and hydroxylated again to the active form, 1,25(OH)D3, mostly in the kidney but also in certain immune cell subsets, by the LDAD risk gene-encoded enzyme Cyp27B1. 1,25(OH)D3 acts as an agonist ligand for the VDR (another MS risk gene), which in turn regulates many pathways, including those in immune response, inflammation, wound healing, and calcium homeostasis, with impact on many cell processes, including immunoregulation [18]. It also controls its own regulation, by upregulating CYP24A1 (also an MS risk gene) on increasing VDR activation [19].

Current treatment of MS with cholecalciferol increases serum 25(OH)D3 levels but does not ameliorate disease [15, 16]. This is surprising, given the many lines of evidence that the vitamin D pathway is underactivated in MS [7, 12, 13], the success of other immunomodulatory agents in treating MS [20], and the reversal of disease on treatment with vitamin D in the mouse models of MS [21]. The vitamin D pathway is under very tight homeostatic control, indicating both its importance and the possibility that intracellular constraints may be limiting the targeted change. Population heterogeneity may be masking successful treatment of a subsection of patients. There are no established biomarkers to assess change within immune cells, or in the immune state, as a result of treatment, including from transcriptomic studies of whole blood [22]. Such markers may indicate successful immune cell modulation, and even a desirable immune response preceding, or more sensitive than, a clinical response. They may also allow stratification of the population to optimise treatment and response. Since vitamin D is likely to be mainly used as an adjunct therapy, here we have compared vitamin D response in MS patients on dimethyl fumarate with controls.

**Fig. 1:**
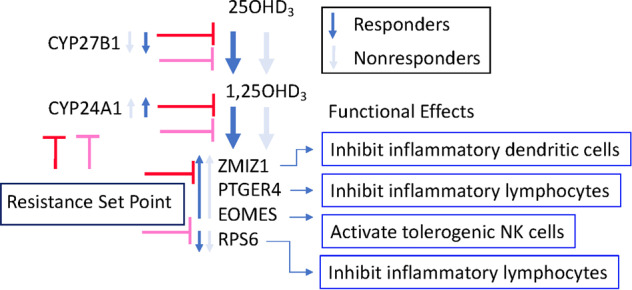
Model of the expected effect of vitamin D responsive MSrisk (VDRMS) genes on response to 25(OH)D3 and on tolerance. Dark blue arrows indicate a larger expected change in responders compared to nonresponders (pale blue arrows).

We reasoned that risk genes regulating and/or responsive to vitamin D may be useful as intracellular immune state markers as they affect pathogenesis. We targeted MS risk genes which are regulated by vitamin D, and with evidence of dysregulation in blood from people with MS. The risk variants of each of these genes are in noncoding gene regions, indicating that the risk variants likely exhibit an effect through regulation of gene expression as opposed to changing the encoded protein [23]. Consequently, their level of activation likely affects pathogenically significant processes. These genes are ZMIZ1, PTGER4, EOMES, CYP24A1, and CYP27B1, collectively called here the VDRMS genes. The expected effect of these VDRMS genes on response to 25(OH)D3 and on tolerance is detailed in Fig. 1 and Supplementary Text. We have tested if the expression of these risk genes might indicate response to vitamin D (25(OH)D3) using models for homeostatic and inflammatory immunological contexts. These data indicate how the expression of these genes might be used to assess response to various forms of vitamin D in clinical trials.

## Materials and methods

### Study design

This study was a cross-sectional in vitro study to assess the response of control and MS patient-derived PBMCs to vitamin D in the presence/absence of different inflammatory stimuli, as detailed further below.

### Source of samples

This study was approved by the Human Research Ethics Committee at Westmead Hospital, and Research Governance at the Westmead Institute for Medical Research under WSLHD HREC Approval 1425. Informed consent was obtained from all subjects. MS patients were diagnosed using the McDonald criteria [24], and were on dimethyl fumarate treatment for a minimum of six months. Blood samples were collected from both MS patients and healthy controls at Westmead Hospital and processed at the Westmead Institute for Medical Research. Healthy controls (HC) were age and gender matched with the patients. Details of the cohorts used in this study are summarised in Table 1. Serum vitamin D (25OHD3) was measured from cryopreserved serum by a pathology provider.

**Table 1:** Details of the cohorts used in the study.

Cohorts		Female	Male	Age (mean ± SD)
Fresh PBMCs (*n* = 10)	Healthy controls	5	5	34.4 ± 8.37
Frozen PBMCs (*n* = 20)	MS DMF patients	8	2	39.2 ± 13.32
	Healthy controls	8	2	40 ± 11.47

### PBMC freeze down procedure

Blood was collected from HC and MS patients in EDTA tubes before being processed in Ficoll Paque Plus (GE Healthcare, Chicago, IL, USA) to isolate PBMCs. Cells were then resuspended in cryopreservation medium (10% dimethyl sulfoxide (DMSO), 50% FBS, RPMI Media) before controlled freezing using a CoolCell (Biocision, San Rafael, CA, USA) and storage at −80 °C prior to use in the study.

### Fresh PBMC cell procedure

Blood samples were collected in EDTA tubes from 10 individuals. Samples were then processed on Ficoll Paque Plus to obtain PBMCs. PBS-washed cells were then suspended in AIMV Serum-Free culture medium (Thermo Fisher, Waltham, MA, USA) to a density of 2 × 10^6^ per ml (vitamin D free media).

### Frozen PBMC cell procedure

Frozen vials of cells were immersed in a 37 °C water bath prior to addition to warmed thawing media (RPMI media, 2% FBS and 10 mM Hepes), followed by multiple washes with PBS to improve cell recovery and viability. Cell pellets were then resuspended and incubated in recovery media (RPMI media, 2% FBS, 1 mM Hepes, 1 mM MgCl_2_, and 200 U/ml DNase I) for 15 min before washing and resting in warmed AIMV media for 24 h at 37 °C and 5% CO_2_. Rested cells were then resuspended in freshly warmed AIMV media to a density of 1.2 × 10^6^–2 × 10^6^ cells per ml.

### Homeostatic protocol

PBMCs (1.2 × 10^5^–2 × 10^5^ cells/well) were plated onto a 96-well U bottom culture plate before the addition of 50 nM Calcifediol (25-hydroxyvitamin D monohydrate, Sigma Aldrich, St Louis, MO, USA) or AIMV media to generate homeostatic conditions with and without vitamin D addition. Cultures were then incubated at 37 °C and 5% CO_2_ for 24 h before harvesting into Cells-to-Signal Lysis Buffer (Thermo Fisher, Waltham, MA, USA) and stored at −80 °C.

### Inflammatory protocol

PBMCs (1.2 × 10^5^–2 × 10^5^ cells/well) were plated on a 96-well U bottom culture plate with various inflammatory stimuli: TNF-α (10ng/ml, Peprotech, Rocky Hill, NJ, USA), ImmunoCult^TM^ Human CD3/CD28 T cell activation beads (CD3/CD28) (as per product sheet, Stem Cell Technologies, Canada), CD40 Ligand (CD40L)(10µg/ml, Sapphire Bioscience) and IFN-β (1000 U/ml, Biogen, Cambridge, MA, USA). Calcifediol (50 nM) or AIMV media were then added to each inflammatory condition before incubation for 24 h at 37 °C and 5% CO_2_. Cells were then harvested into Cells-to-Signal Lysis buffer and stored at −80 °C.

### RNA extraction, cDNA synthesis, and gene expression analysis

Samples stored at −80 °C were thawed and prepared for RNA extraction as per instructions in the Isolate II Micro RNA Extraction Kit (Bioline, United Kingdom). This was followed by cDNA synthesis using SuperScript® IV (Thermo Fisher, Waltham, MA). Gene expression was then determined by real-time polymerase chain reaction (RT-PCR) using TaqMan Universal Master Mix II (Catalog No. 4304437, Life Technologies, Carlsbad, CA) and the following TaqMan gene expression assays (CYP27B1: Hs01096154_m1, CYP24A1: Hs00167999_m1, ZMIZ1:Hs00393480_m1, EOMES: Hs00172872_m1, PTGER4: Hs00168781_m1, RPS6: Hs04195024_g1, RPL30: Hs00265497_m1 (Life Technologies, Carlsbad, CA). Relative expression to the housekeeping gene, RPL30, was then determined.

### Statistical analysis

Data were analysed using GraphPad Prism 8 (GraphPad Software, La Jolla, CA). To compare between nonpaired samples, the Mann–Whitney test was used. For paired samples, the Wilcoxon signed-rank test was used. Correlations were calculated using Spearman’s rank correlation. To minimise type two errors, no correction for multiple testing was performed on the *p* values obtained in each of the tests, with a *p* value of <0.05 considered significant.

## Results

### Response to vitamin D: homeostatic model

**Fig. 2:**
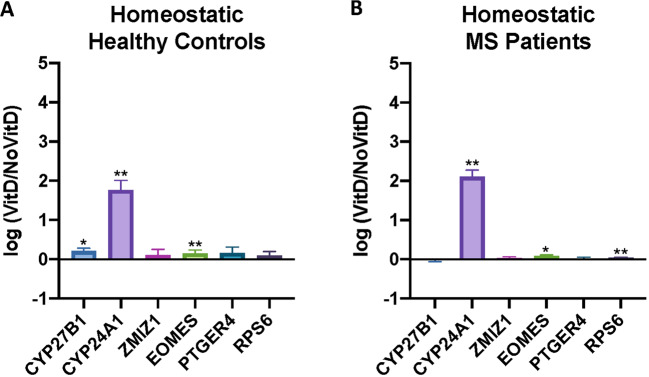
Gene expression of MS risk genes in PBMCs on culture with calcefidiol (VitD). **A** cryopreserved PBMCs, controls, and **B** cryopreserved PBMCs from MS patients. Wilcoxon signed-rank tests were conducted to obtain *p* values represented by asterisks: *p < 0.05, **p < 0.01, ***p < 0.001, and ****p < 0.0001.

For comparing cohorts of collected samples from clinical trials, it is more practical to store PBMCs as cryopreserved samples for later testing. We therefore compared the response of PBMCs thawed from cryopreserved samples treated for 24 h with and without 50 nM of Calcifediol (25(OH)D3), called here the homeostatic model. (Fig. 2A, B). Our small cohort size was powered only to detect large effects, as required for biomarker utility. CYP27B1 expression increased for healthy controls (*p* = 0.0156, Wilcoxon signed-rank test), with CYP24A1 expression increasing for both healthy controls and MS (*p* = 0.0039). EOMES expression was also significantly increased on 25(OH)D3 incubation for healthy controls and MS (*p* = 0.002, *p* = 0.0195, respectively). RPS6 expression was also significantly increased for MS (*p* = 0.0195).

We also assessed changes in expression of the VDRMS genes in fresh ex vivo PBMCs treated with vitamin D (Supplementary Fig. 1). The response was more marked, and again the expression of CYP24A1 increased significantly (*p* = 0.0039), and RPS6 decreased (*p* = 0.0039). Expression of the other VDRMS genes was not significantly affected by vitamin D.

### Response to vitamin D: inflammatory model

As MS is considered to represent an inflammatory condition [25], we then devised a test for response to 25(OH)D3 of PBMCs in the presence of inflammatory agents. The major immune cell subsets of PBMCs are T cells, B cells, monocytes, and natural killer cells. We used standard inflammatory agents to target these subsets: antiCD3/antiCD28 to stimulate T cells, CD40L to stimulate B cells, TNF-α to target monocytes, and IFN-β to target NK cells. Most of these agents will also have pan-immune cell subset effects.

**Fig. 3:**
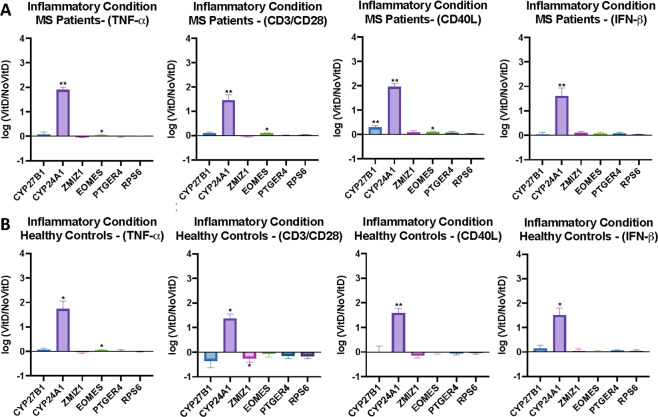
Gene expression of MS risk genes in PBMCs on culture with calcefidiol and inflammatory agents. **A** Cryopreserved PBMCs, MS patients, and **B** cryopreserved PBMCs from healthy controls. Wilcoxon signed-rank tests were performed to analyze vitamin D effects within each inflammatory condition. Asterisks are indicative of *p* values: *p < 0.05, **p < 0.01, ***p < 0.001 and ****p < 0.0001.

For the cryopreserved PBMCs from HC, using the inflammatory model, CYP24A1 expression again increased significantly in all conditions. EOMES and ZMIZ1 showing altered expression (increased EOMES for TNF-α, *p* = 0.049; decreased ZMIZ1 for CD3/CD28, *p* = 0.027, Wilcoxon signed-rank test) (Fig. 3B). A similar pattern was observed for MS, notably, that CYP24A1 levels also increased for MS (Fig. 3A). For EOMES, expression levels increased in more conditions compared to control, again with TNF-α (*p* = 0.037), as well as antiCD3/CD28 (*p* = 0.0195) and CD40L (*p* = 0.0195) (Fig. 2C). CD40L stimulation resulted in a significant increase in expression of CYP27B1 in the MS samples (*p* = 0.002) but this was not observed in the HC.

Since CYP27B1 activates 25(OH)D3 and CYP24A1 inactivates the activated form, 1,25(OH)D3; the ratio of CYP27B1/CYP24A1 should indicate a combined effect of the two genes. This ratio was significantly lower on the addition of vitamin D in the homeostatic model (*p* = 0.0039) and inflammatory models (*p* = 0.0156–0.002) (Supplementary Fig. 2A). A similar pattern was observed using cryopreserved PBMCs for controls and MS (Supplementary Fig. 2B, C).

### Do serum 25(OH)D3 levels predict changes in the expression of VDRMS genes?

**Fig. 4:**
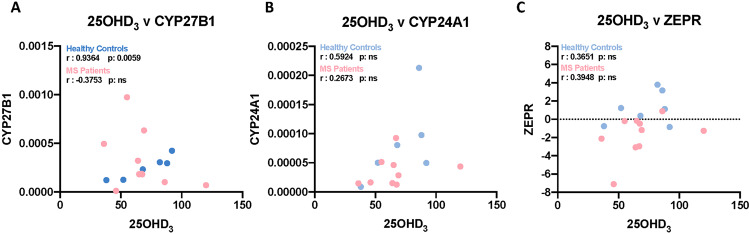
Correlation between Serum 25OHD_3_ with CYP27B1, CYP24A1, and ZEPR expression. Spearman’s rank correlation was performed to obtain *r* and *p* values for both healthy controls and MS patients.

Serum 25(OH)D3 levels were not different between the MS patients (many on cholecalciferol supplementation) and controls, mean of 67.5 nmol/L for MS (range 36–120); 69 nmol/L for controls (range 38–92). There was an indication that 25(OH)D3 levels affected CYP27B1 for controls (*r* = 0.93, *p* = 0.0059, Spearman’s rank correlation), but not for the other genes (Fig. 4).

### Response of MS patients PBMCs compared to healthy controls

**Fig. 5:**
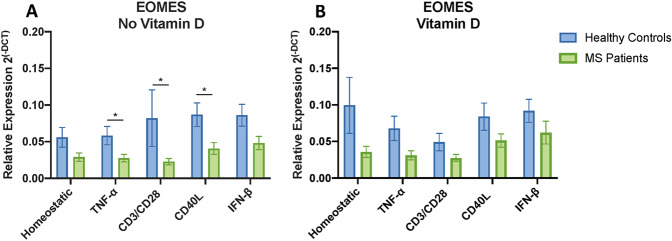
Comparison of vitamin D response in PBMCs from healthy controls and PWMS for EOMES. Mann–Whitney tests were performed for each inflammatory condition, with (**A**) no vitamin D and (**B**) vitamin D. *P* values obtained by statistical analysis are represented by asterisks: *p < 0.05, **p < 0.01, ***p < 0.001, and ****p < 0.0001.

**Fig. 6:**
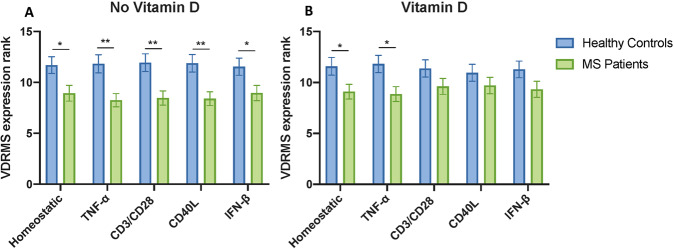
The combined ranks of gene expression for VDRMS genes are lower for MS compared to HC without vitamin D, but less so with vitamin D, across conditions. Mann–Whitney tests were performed for each inflammatory condition, with (**A**) no vitamin D and (**B**) vitamin D. *P* values obtained by statistical analysis are represented by asterisks: *p < 0.05, **p < 0.01, ***p < 0.001, and ****p < 0.0001.

Since patients with MS (PWMS) have evidence of a less functional vitamin D pathway, we expected their immune cells would have an altered response to vitamin D compared to HC. As well as comparing the pattern of vitamin D response for MS and HC for the VDRMS genes (previous sections), we also compared MS directly to healthy controls for VDRMS gene expression in each condition. Our small cohort size was powered only to detect large effects, as required for biomarker utility. In the absence of vitamin D, only EOMES expression (and PTGER4 for some) was different between MS and HC, reduced in some inflammatory conditions for MS (Fig. 5A). With vitamin D treatment (Fig. 5B), the initial pattern observed in EOMES is absent for most conditions. Notably, there was a trend of decreased expression for most of the VDRMS genes in MS across all the homeostatic and inflammatory conditions (Mann–Whitney test, Supplementary Figs. 3–4). This decrease was significant when compared collectively (using ranks of gene expression for each gene, for HC and MS, all genes included, rather than just the ranks of one gene at a time) for the homeostatic (*p* = 0.0172), and inflammatory models (*p* = 0.027–0.0022) (Fig. 6).

### Starting gene expression levels correlate with response levels across the conditions

**Fig. 7:**
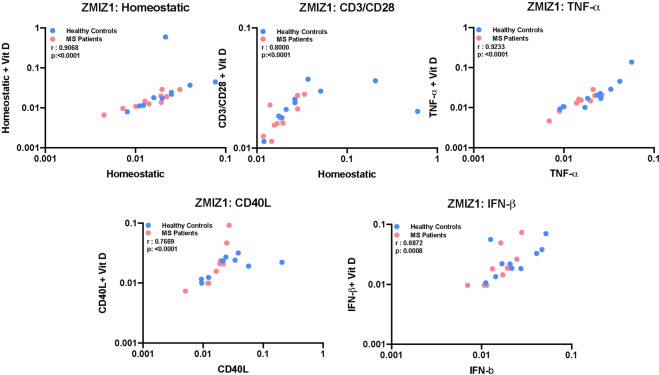
Correlation between ZMIZ1 expression level for vitamin D and no vitamin D conditions in both homeostatic and inflammatory contexts. Spearman’s rank correlation was performed to obtain *r* and *p* values. A summary of correlation results performed on the other genes are reported in Supplementary Table 1.

To determine if expression without treatment correlated with an expression on treatment with vitamin D, in the homeostatic and inflammatory models, we ranked the expression level of the genes within the total cohort and then calculated the Spearman’s rank correlation and corresponding *p* values for starting levels compared to response levels. Significant *p* values mean they do correlate. For the majority of genes, there was a significant correlation between their ranks in the starting and response conditions, usually with a *p* < 0.0001 (Fig. 7 for ZMIZ1, Supplementary Table 1 for all). The exception was CYP24A1.

### Gene regulatory networks in both models are more correlated in healthy controls than PWMS

In transcriptomic studies of whole blood in Australian and International cohorts, we had identified sets of genes with highly correlated expression, tagged by ZMIZ1 [26] and EOMES [27]. RPS6 was highly negatively correlated with ZMIZ1. EOMES, and ZMIZ1 expression was weakly correlated. This suggested co-regulation at the transcriptomic/immunologic/immune cell subset level. Expression of CYP27B1 and CYP24A1 were not detectable in these whole blood cohorts. In our PBMC dataset, we tested if the expression of the VDRMS genes were correlated with each other by comparing their ranks using Spearman’s rank correlation (Supplementary Fig. 5). In the absence of vitamin D, there was a significant correlation between CYP27B1/ZMIZ1, CYP24A1/PTGER4, and ZMIZ1/RPS6 (all *R* > 0.46). PTGER4 and EOMES were very tightly correlated (*R* = 0.89, *p* < 7 × 10^−8^). The between gene correlations were much stronger in controls compared to PWMS. On culture with vitamin D in the controls, the correlation between CYP27B1, CYP24A1, ZMIZ1; and between PTGER4 and EOMES was highly significant (*R* > 0.8, *p* < 0.01), but only the latter was significant in MS.

## Discussion

We show here that the expression of MS risk genes is affected by the addition of 25(OH)D3 in culture in homeostatic and inflammatory models, but that it was not significantly correlated with serum 25(OH)D3 levels in the donors. The generesponsible for reducing intracellular levels of activated vitamin D, CYP24A1, has increased expression in peripheral blood cells (PBMC) treated with 25(OH)D3. It also increases in expression when PBMCs are treated with inflammatory agents. Expression of the gene responsible for activating 25(OH)D3 to 1,25(OH)D3, CYP27B1, was also altered on treatment with 25(OH)D3 over the tested period (24 h) in many conditions. The immunological benefit of changes in CYP24A1 and CYP27B1 expression is difficult to assess. Increased expression of CYP24A1 may indicate homeostatic response to increased production/availability of activated vitamin D; but it could also indicate reduced response to 1,25(OH)D3 due to increased catabolism. Increased CYP27B1 gene expression might be expected in response to increasing substrate (25(OH)D3), and decreased CYP27B1 could be a response to increased 1,25(OH)D3. Consequently, expression of neither gene would necessarily indicate useful downstream changes to the immune state, and also would not be useful in comparing 25(OH)D3 with 1,25(OH)D3 preparations. The other MS risk genes would be potentially more useful for both purposes.

Of the other MS risk genes assayed, EOMES showed evidence of vitamin D response, albeit at a reduced level compared to CYP24A1, in various conditions. Expression levels of many of the MS risk genes were also correlated with each other, dependent on treatment and disease. Starting expression level of the VDRMS genes predicted response level, consistent with the concept that there are interindividual differences in settings for vitamin D response. In cells from PWMS (all on dimethyl fumarate, most with cholecalciferol supplementation) expression of VDRMS genes was lower in the absence of vitamin D in both models (by rank test), but this difference largely disappeared on vitamin D addition. This is consistent with a beneficial effect mediated by these genes in response to vitamin D. There was a high correlation in expression observed between many VDRMS genes in PBMCs from controls, but mostly not for PBMCs from PWMS. This may be due to dysregulation of the transcription of these genes in PBMCs in MS. Increasing the VDRMS gene expression, and restoring the correlation between genes, by vitamin D treatment may be evidence of a beneficial immune effect.

The evidence here is that PWMS on dimethyl fumarate and supplementation have different expression and regulation of the VDRMS genes, a different response of these genes to vitamin D and an abrogation of the differences in the absence of vitamin D upon vitamin D treatment. This is despite the expected shift towards a tolerogenic phenotype previously described for this treatment [28]. Our data suggest a further tolerogenic shift may be possible towards the control phenotype with improved vitamin D regulation of the VDRMS genes.

Replication of our findings using separate cohorts, cohorts on different therapies, and untreated cohorts is needed. Also, larger cohorts will improve sensitivity to identifying gene expression responses of the biomarkers. The effect of vitamin D on immune cell gene expression in vivo may be different from our findings, both immediately after supplementation and long term, due to homeostatic regulation and cross-talk of vitamin D pathway regulatory factors between different tissues. The pathogenically significant changes to immunological state which follow changes in VDRMS gene expression will ultimately only be known from clinical studies. Risk genotype effects on expression and vitamin D response have previously been reported by us [26, 29]. These are too small for utility as biomarkers, but important in establishing the pathogenic significance of the risk loci. These risk SNP effects need to be determined for relevant immunological contexts, such as the two models used here.

Studies to distinguish between association and causation are ongoing, but the evidence for the latter is now quite strong [30]. Further, the evidence that the vitamin D pathway is suboptimal in MS comes not only from the latitude effect, reduced levels of serum 25(OH)D3 seen in untreated MS patients, particularly associated with relapses and other clinical markers [31]; and the mouse studies indicating the reversal of disease with vitamin D amelioration [21]; but now also from the genetic studies identifying the genes regulating, and regulated by vitamin D, as risk factors [12]. It is too soon to disregard the potential benefit of ameliorating vitamin D in MS, and new tools such as the potential biomarkers identified here may be useful in future studies.

## Conclusion

Expression of VDRMS genes changes in models of homeostasis and inflammation, in several cases differently between PWMS and controls. As these genes are pathogenically significant in MS (MS risk genes) and their expression is correlated with each other in many experimental conditions and in vivo, the change in expression may correspond to the altered immune cell state that indicates a protective effect of vitamin D, i.e., the therapeutic benefit. As such, these data also suggest these genes are useful biomarkers for use in assessing the utility of vitamin D analogues for the treatment of MS, and for establishing clinically important population heterogeneity affecting vitamin D response.

## Supplementary information

Supp Table and Supp Figure legends

Supplementary Text

Supplementary Figure 1

Supplementary Figure 2

Supplementary Figure 3

Supplementary Figure 4

Supplementary Figure 5
