# Supplementary material for: Expression of CYP24A1 and other multiple sclerosis risk genes in peripheral blood indicates response to vitamin D in homeostatic and inflammatory conditions
Source: Genes Immun. 2021 Jun 23;22(4):227–33. doi: 10.1038/s41435-021-00144-6 (PMC8387232; doi:10.1038/s41435-021-00144-6)
Supplement: Supplementary file 1 — Supp Table and Supp Figure legends [file 41435_2021_144_MOESM1_ESM.docx]

**Supplementary Text**

Rationale for Biomarker Selection

**Supplementary Figure 1**

*Gene expression of MS risk genes in Freshly isolated PBMCs on culture with calcefidiol (VitD) in controls.* Wilcoxon signed-rank tests were conducted to obtain p values represented by asterisks: * p<0.05, ** p<0.01, *** p<0.001 and **** p<0.0001.

**Supplementary Figure 2**

*Ratio of expression of CYP24A1 and CYP27B1 in freshly isolated and cryopreserved PBMCs.*

Wilcoxon signed-rank tests were performed between vitamin D and no vitamin D groups, with asterisks indicating p values: * p<0.05, ** p<0.01, *** p<0.001 and **** p<0.0001.

**Supplementary Figure 3**

*Comparison of gene expression differences in PBMCs from healthy controls and PWMS for CYP27B1, CYP24A1, PTGER4, ZMIZ1, RPS6 and the CYP27B1/CYP24A1 ratio.*

Mann-Whitney tests were performed for each condition, without vitamin D treatment. P values obtained by statistical analysis are represented by asterisks: * p<0.05, ** p<0.01, *** p<0.001 and **** p<0.0001.

**Supplementary Figure 4**

*Comparison of gene expression differences in vitamin D treated PBMCs from healthy controls and PWMS for CYP27B1, CYP24A1, PTGER4, ZMIZ1, RPS6 and the CYP27B1/CYP24A1.*

Mann-Whitney tests were performed for each condition, with vitamin D treatment. P values obtained by statistical analysis are represented by asterisks: * p<0.05, ** p<0.01, *** p<0.001 and **** p<0.0001.

**Supplementary Figure 5**

*Correlation of expression of VDRMS genes with each other.* Spearman’s rank correlation analysis was used to obtain p values.

**Supplementary Table 1**

*P values for comparison of starting levels to response levels to vitamin D/inflammatory stimuli in HC and MS.* Spearman’s rank correlation p values are reported. ns = p>0.05

|  |  |  |  |  |  |  |
| --- | --- | --- | --- | --- | --- | --- |
| **HC + MS** | **CYP27B1** | **CYP24A1** | **EOMES** | **ZMIZ1** | **PTGER4** | **RPS6** |
| **Homeostatic v Homeostatic + D** | <0.0001 | ns | <0.0001 | <0.0001 | <0.0001 | <0.0001 |
| **TNFa v TNFa + D** | <0.0001 | ns | <0.0001 | <0.0001 | <0.0001 | <0.0001 |
| **CD3/CD28 vs CD3/CD28 + D** | 0.0014 | ns | <0.0001 | <0.0001 | 0.0005 | 0.0455 |
| **CD40L v CD40L + D** | <0.0001 | ns | <0.0001 | <0.0001 | 0.005 | <0.0001 |
| **IFNb vs IFNb + D** | 0.0002 | ns | <0.0001 | <0.0004 | <0.0001 | 0.0088 |
| **Homeostatic v TNFa** | 0.0001 | 0.0868 | <0.0001 | <0.0001 | <0.0001 | <0.0001 |
| **Homeostatic v CD3/CD28** | 0.0527 | ns | <0.0001 | <0.0002 | 0.0246 | ns |
| **Homeostatic v CD40L** | <0.0001 | 0.008 | <0.0001 | <0.0001 | <0.0001 | <0.0001 |
| **Homeostatic v IFNb** | <0.0001 | 0.0286 | <0.0001 | <0.0001 | <0.0001 | <0.0001 |
|  |  |  |  |  |  |  |
| **HC** | **CYP27B1** | **CYP24A1** | **EOMES** | **ZMIZ1** | **PTGER4** | **RPS6** |
| **Homeostatic v Homeostatic + D** | 0.0006 | ns | 0.0011 | 0.0014 | ns | 0.0014 |
| **TNFa v TNFa + D** | <0.0001 | ns | <0.0001 | <0.0001 | <0.0001 | <0.0001 |
| **CD3/CD28 vs CD3/CD28 + D** | ns | ns | 0.0117 | 0.0173 | ns | ns |
| **CD40L v CD40L + D** | 0.0006 | ns | 0.0063 | ns | ns | 0.0044 |
| **IFNb vs IFNb + D** | 0.0053 | ns | <0.0001 | ns | 0.0004 | ns |
| **Homeostatic v TNFa** | 0.0041 | ns | <0.0001 | 0.0063 | 0.0367 | 0.0029 |
| **Homeostatic v CD3/CD28** | ns | ns | 0.0173 | 0.0134 | ns | ns |
| **Homeostatic v CD40L** | 0.001 | ns | 0.0024 | 0.0087 | 0.0014 | 0.0014 |
| **Homeostatic v IFNb** | 0.0001 | ns | 0.0245 | 0.0063 | 0.0101 | 0.0063 |
|  |  |  |  |  |  |  |
| **MS** | **CYP27B1** | **CYP24A1** | **EOMES** | **ZMIZ1** | **PTGER4** | **RPS6** |
| **Homeostatic v Homeostatic + D** | <0.0001 | ns | <0.0001 | 0.0011 | 0.0003 | 0.0075 |
| **TNFa v TNFa + D** | 0.0053 | ns | <0.0001 | 0.0003 | 0.0036 | 0.0011 |
| **CD3/CD28 vs CD3/CD28 + D** | 0.0014 | ns | 0.0001 | 0.0173 | <0.0001 | ns |
| **CD40L v CD40L + D** | 0.0044 | ns | 0.0006 | 0.0004 | 0.0334 | 0.0011 |
| **IFNb vs IFNb + D** | ns | ns | <0.0001 | 0.0019 | 0.0245 | ns |
| **Homeostatic v TNFa** | 0.0195 | 0.0109 | <0.0001 | <0.0001 | 0.0036 | <0.0334 |
| **Homeostatic v CD3/CD28** | ns | 0.0023 | 0.0014 | 0.0117 | ns | ns |
| **Homeostatic v CD40L** | 0.0014 | 0.0481 | <0.0001 | 0.0004 | 0.0002 | 0.0334 |
| **Homeostatic v IFNb** | 0.0019 | ns | <0.0001 | 0.0019 | 0.0101 | 0.0063 |
