# Supplementary material for: Expression of CYP24A1 and other multiple sclerosis risk genes in peripheral blood indicates response to vitamin D in homeostatic and inflammatory conditions
Source: Genes Immun. 2021 Jun 23;22(4):227–33. doi: 10.1038/s41435-021-00144-6 (PMC8387232; doi:10.1038/s41435-021-00144-6)
Supplement: Supplementary file 2 — Supplementary Text [file 41435_2021_144_MOESM2_ESM.docx]

**Supplementary Text**

Rationale for Biomarker Selection

We had earlier found that ZMIZ1, PTGER4 and EOMES were underexpressed in blood in MS, replicated in multiple cohorts from Australia and the USA, and that their expression is correlated with each other (1, 2). ZMIZ1 and EOMES tag separate gene modules (1, 2), but show weak correlation with each other. The gene PTGER4 is in the ZMIZ1 gene module, and is a risk gene for a similar set of autoimmune diseases as ZMIZ1 (3). The ZMIZ1 gene encodes Zinc Finger MIZ-Type Containing 1, a member of the protein inhibitor of activated STAT (PIAS)-like family of coregulators, thought to bind and regulate other DNA-binding transcription factors. Its higher expression in immature and tolerogenic myeloid cells and increase on vitamin D stimulation in these cells suggests it prevents differentiation of inflammatory dendritic cells (1). PTGER4 (prostaglandin E receptor 4) is a receptor for the UVB sensitive agent prostaglandin E2, known to slow disease in EAE (4). It may prevent lymphocyte proliferation (5). EOMES encodes Eomesodermin, a transcription factor which regulates genes across many immune cell types (2). It may increase activation of Natural Killer cells preventing autoimmunity (2). Recently we have shown that CYP24A1, ZMIZ1 and PTGER4 have VDR binding peaks within 5kb of the gene transcription start sites (6). CYP27B1 catalyses the addition of a hydroxyl group to 25(OH)D3, making it 1,25(OH)D3, enabling its activation of the vitamin D receptor. CYP24A1 catabolises 1,25(OH)D3, rendering it inactive. Expression of CYP24A1 and CYP27B1 is very low in blood, not readily measured by standard transcriptomic approaches, and association of expression with disease is not yet reported. We also investigated the gene RPS6, previously shown to be overexpressed in MS (7), affected by season, and its protein is phosphorylated by the MS risk gene RPS6KB1. Lower expression of RPS6 may correspond to reduced lymphocyte activation (7).

1. Fewings NL, Gatt PN, McKay FC, Parnell GP, Schibeci SD, Edwards J, et al. The autoimmune risk gene ZMIZ1 is a vitamin D responsive marker of a molecular phenotype of multiple sclerosis. J Autoimmun. 2017;78:57-69.

2. McKay FC, Gatt PN, Fewings N, Parnell GP, Schibeci SD, Basuki MA, et al. The low EOMES/TBX21 molecular phenotype in multiple sclerosis reflects CD56+ cell dysregulation and is affected by immunomodulatory therapies. Clin Immunol. 2016;163:96-107.

3. Carvalho-Silva D, Pierleoni A, Pignatelli M, Ong C, Fumis L, Karamanis N, et al. Open Targets Platform: new developments and updates two years on. Nucleic Acids Res. 2018.

4. Esaki Y, Li Y, Sakata D, Yao C, Segi-Nishida E, Matsuoka T, et al. Dual roles of PGE2-EP4 signaling in mouse experimental autoimmune encephalomyelitis. Proc Natl Acad Sci U S A. 2010;107(27):12233-8.

5. Murn J, Alibert O, Wu N, Tendil S, Gidrol X. Prostaglandin E2 regulates B cell proliferation through a candidate tumor suppressor, Ptger4. J Exp Med. 2008;205(13):3091-103.

6. Booth DR, Ding N, Parnell GP, Shahijanian F, Coulter S, Schibeci SD, et al. Cistromic and genetic evidence that the vitamin D receptor mediates susceptibility to latitude-dependent autoimmune diseases. Genes Immun. 2016;17(4):213-9.

7. Parnell GP, Gatt PN, McKay FC, Schibeci S, Krupa M, Powell JE, et al. Ribosomal protein S6 mRNA is a biomarker upregulated in multiple sclerosis, downregulated by interferon treatment, and affected by season. Multiple sclerosis (Houndmills, Basingstoke, England). 2014;20(6):675-85.
