# Supplementary figures and images for: Expression of CYP24A1 and other multiple sclerosis risk genes in peripheral blood indicates response to vitamin D in homeostatic and inflammatory conditions
Source: Genes Immun. 2021 Jun 23;22(4):227–33. doi: 10.1038/s41435-021-00144-6 (PMC8387232; doi:10.1038/s41435-021-00144-6)

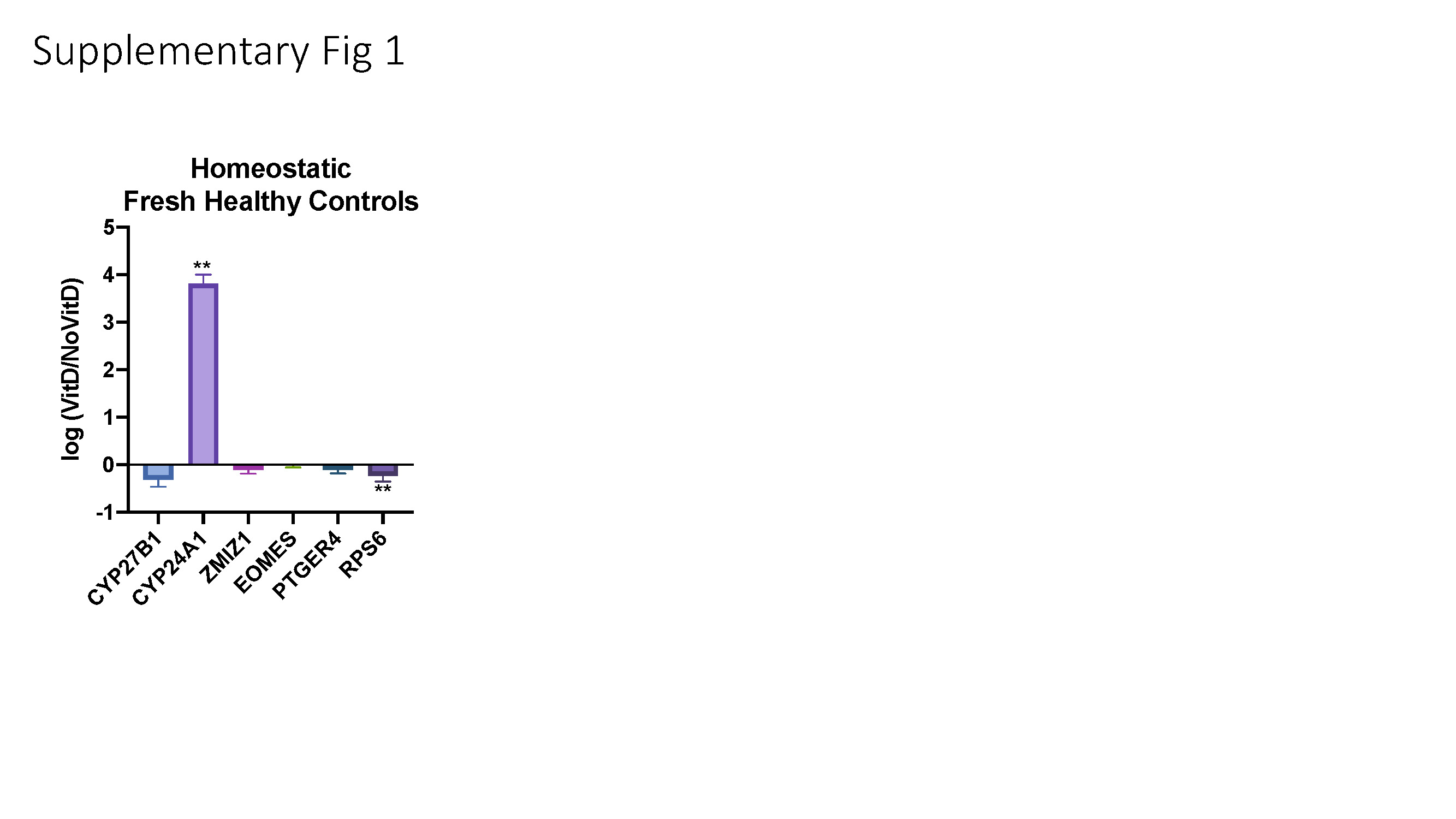

Supplement: Supplementary file 3 — Supplementary Figure 1 [file 41435_2021_144_MOESM3_ESM.jpg]

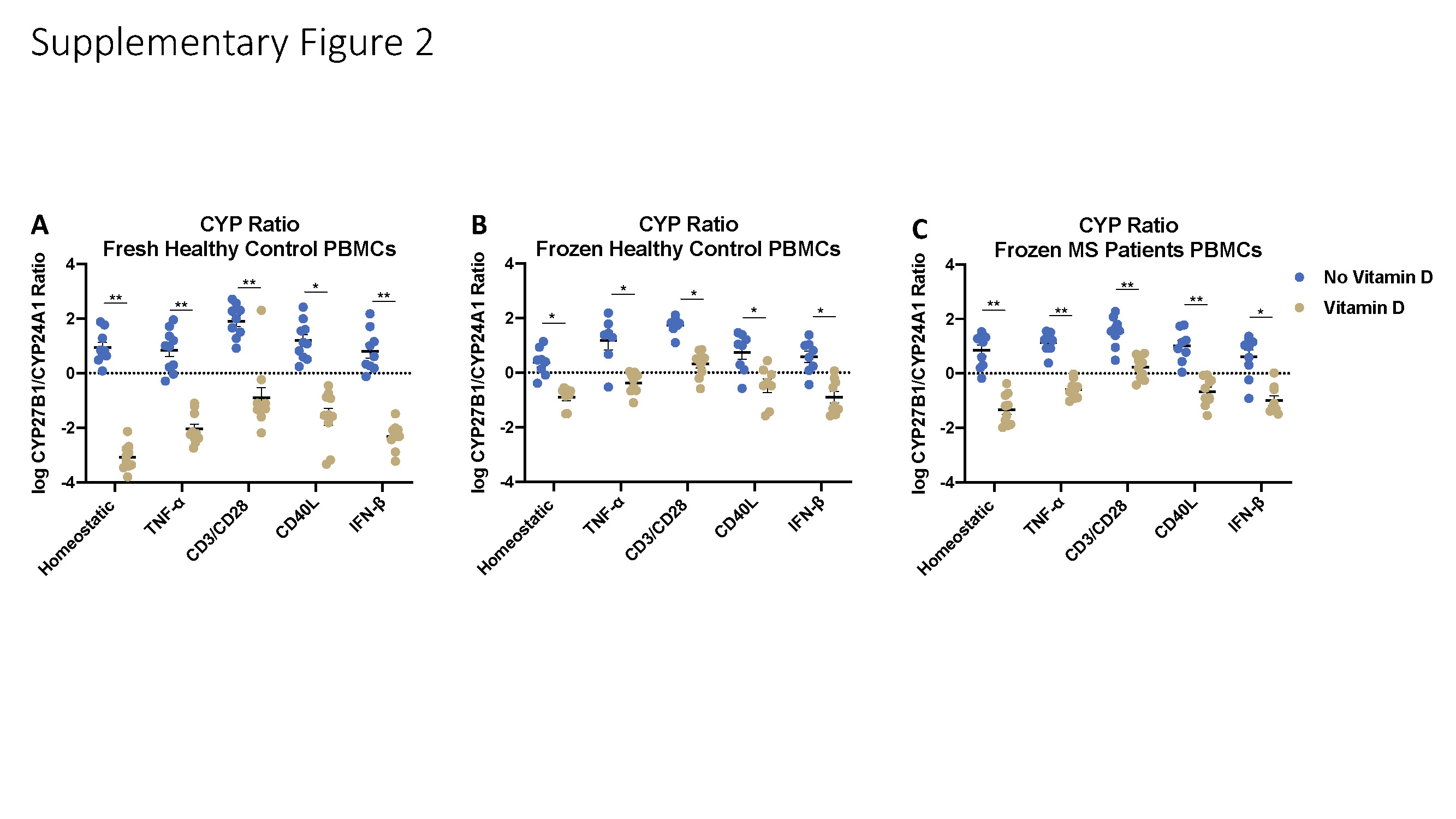

Supplement: Supplementary file 4 — Supplementary Figure 2 [file 41435_2021_144_MOESM4_ESM.jpg]

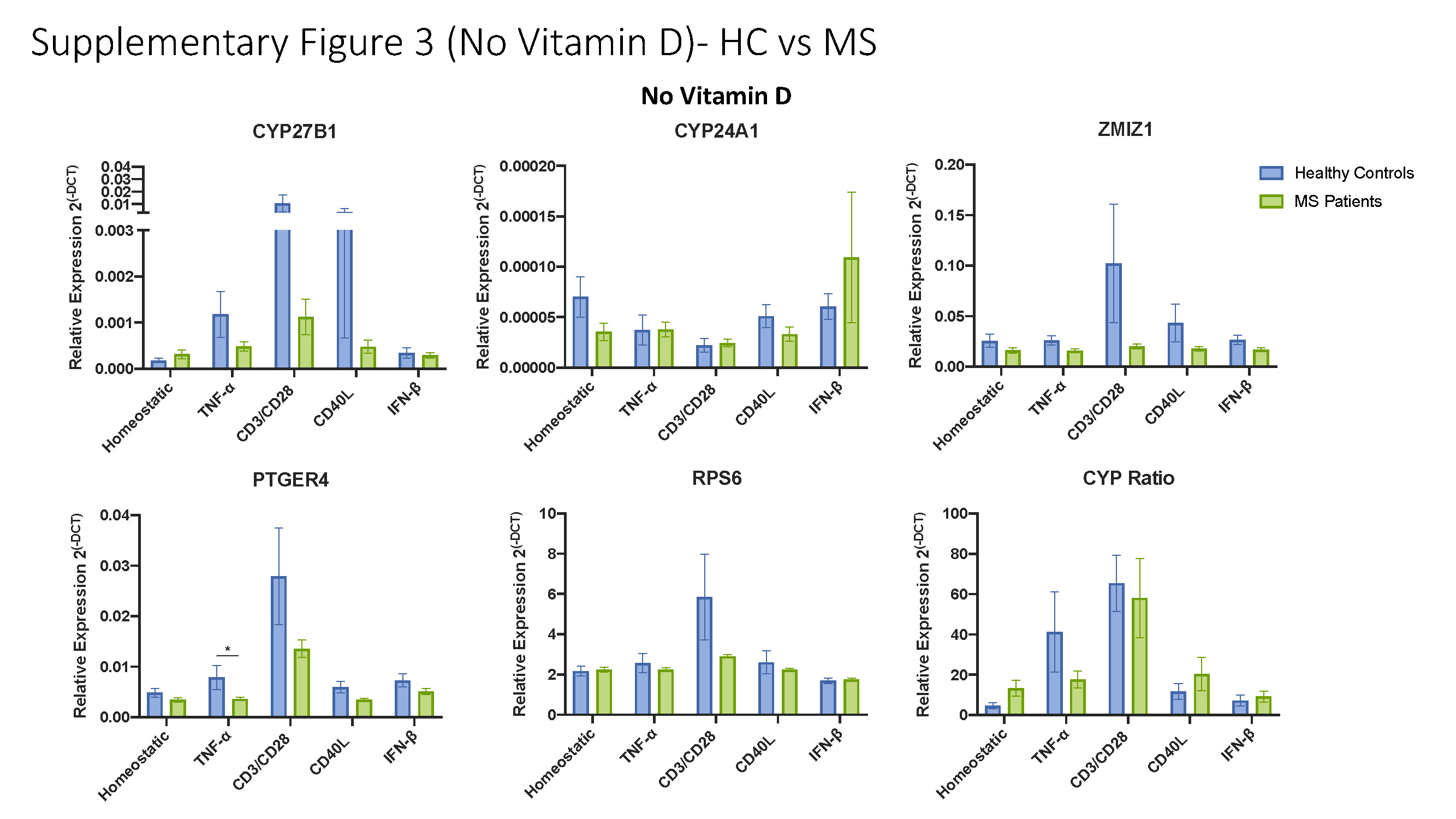

Supplement: Supplementary file 5 — Supplementary Figure 3 [file 41435_2021_144_MOESM5_ESM.jpg]

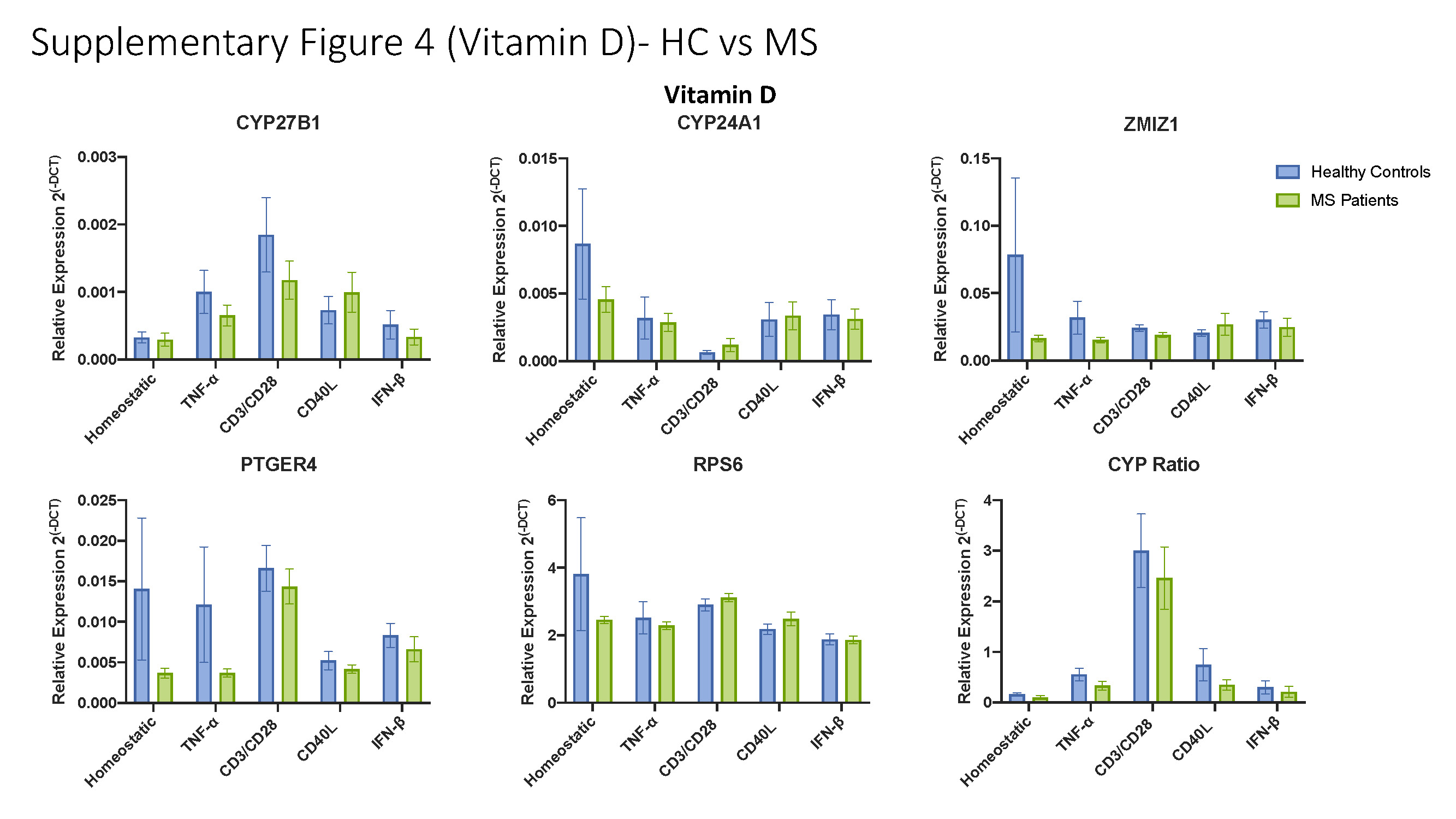

Supplement: Supplementary file 6 — Supplementary Figure 4 [file 41435_2021_144_MOESM6_ESM.jpg]

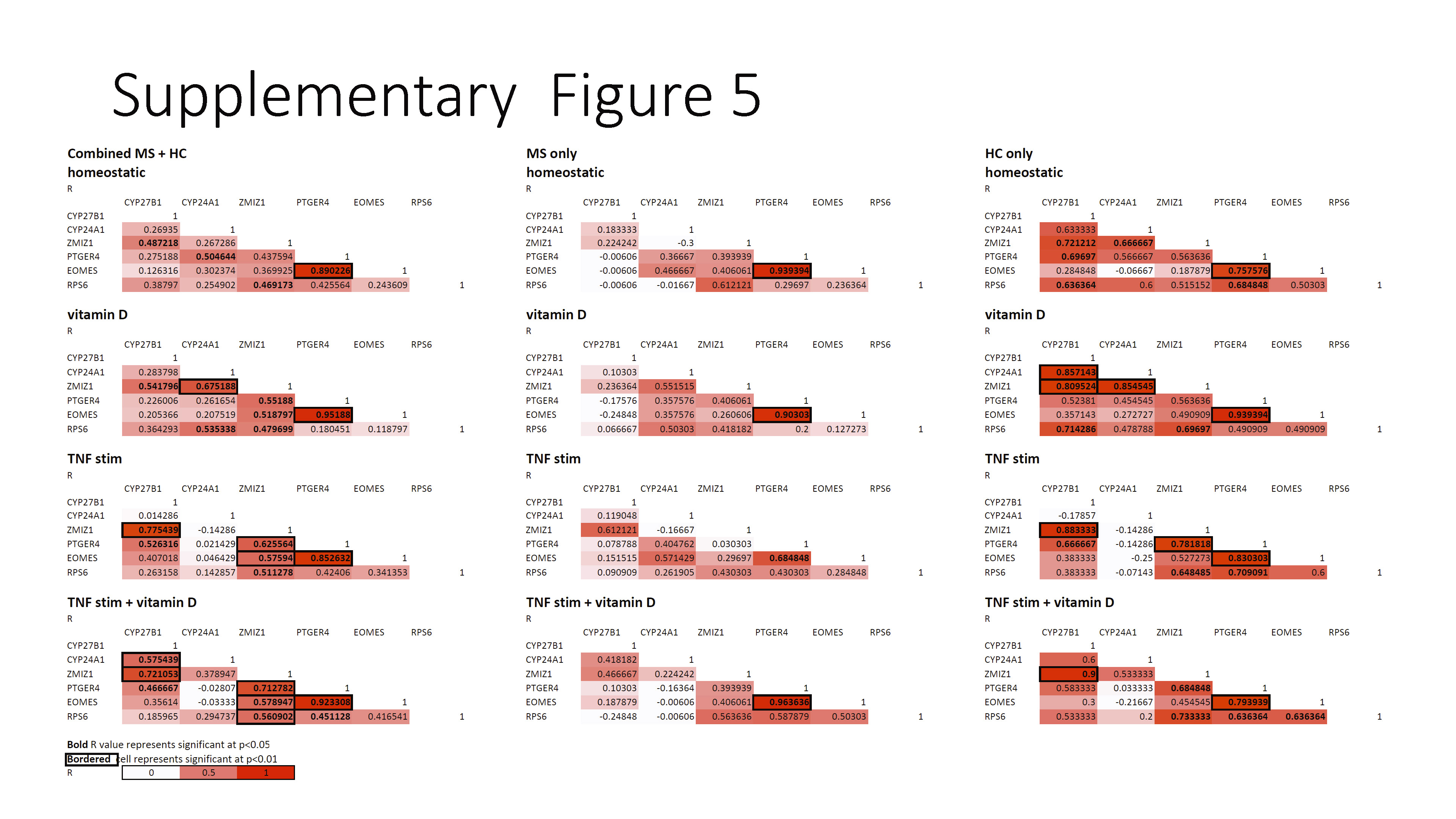

Supplement: Supplementary file 7 — Supplementary Figure 5 [file 41435_2021_144_MOESM7_ESM.jpg]
